# Elevated Mitochondrial Reactive Oxygen Species within Cerebrospinal Fluid as New Index in the Early Detection of Lumbar Spinal Stenosis

**DOI:** 10.3390/diagnostics11050748

**Published:** 2021-04-22

**Authors:** Jin Young Hong, Hyunseong Kim, Wan-Jin Jeon, Junseon Lee, In-Hyuk Ha

**Affiliations:** Jaseng Spine and Joint Research Institute, Jaseng Medical Foundation, Seoul 135-896, Korea; vrt3757@gmail.com (J.Y.H.); biology4005@gmail.com (H.K.); poghkl@gmail.com (W.-J.J.); excikind@gmail.com (J.L.)

**Keywords:** cerebrospinal fluid, lumbar spinal stenosis, mitochondria, reactive oxygen species

## Abstract

Lumbar spinal stenosis (LSS) is a common neurodegenerative condition. However, how neurogenic claudication develops with severe leg pain has not yet been clearly elucidated. Moreover, cerebrospinal fluid (CSF) physiology at the lumbosacral level is poorly understood because of the difficulties involved in quantification and visualization. Recent studies have suggested that assessment of mitochondrial function in CSF provides an indirect way to assess neurological disorders and an important feature of disease progression. In this study, we assessed the relevance of endogenous extracellular mitochondria in the CSF of rats after LSS. Mitochondrial changes within the CSF were analyzed following LSS at 1 week using flow cytometry. An increase in cell size and number was observed in CSF with LSS, and reactive oxygen species (ROS) levels were also increased within the CSF at 1 week in the LSS group. Elevated mitochondrial ROS and functional changes in the CSF are hallmarks of LSS. The present study is the first to demonstrate that elevated mitochondrial ROS within the CSF is a new index for the early detection of LSS. Moreover, it may represent a potential novel treatment target for LSS.

## 1. Introduction

Cerebrospinal fluid (CSF) can be used for the early diagnosis of changed pathophysiological states of various conditions and as a therapeutic indicator [1]. Existing clinical studies have reported a correlation between CSF dynamics and the pathogenesis of lumbar spinal stenosis (LSS). A slower CSF flow was observed in patients with LSS than in healthy volunteers [2,3,4]. Specifically, the sacral CSF flow after walking was barely detectable in LSS patients, and the peak flow velocity was slower in LSS patients than healthy controls. This change in CSF dynamics can be suggested as a test method for early diagnosis in the prediction of neurological signs of LSS, including claudication, but it is not specific enough to present a therapeutic target and is not sufficient as relevant evidence. In recent years, studies on neurological diseases related to CSF have mainly analyzed mitochondria [5,6,7,8]. In recent studies, it has been reported that intercellular transfer of mitochondria is possible, and when cells are damaged and function is impaired, mitochondria from other cells are released into the extracellular space and transported to rescue and restore the damaged cells [9,10]. In addition, it was reported that mitochondria released into the extracellular space were found in the CSF, and morphological and functional changes of mitochondria in CSF were related to disease progression and therapeutic course [11,12]. A study that comparatively analyzed the functional relevance of endogenous extracellular mitochondria in human and rat CSF after subarachnoid hemorrhage (SAH) also showed that the mitochondrial membrane potential (MMP) of mitochondria in CSF decreased after SAH compared to the control group. In addition, extracellular mitochondria were detected in human CSF, and MMP decreased after SAH. Their results showed that a higher MMP in mitochondria within the CSF was correlated with good clinical recovery 3 months after the onset of SAH [5]. As can be seen from these previous studies, mitochondrial changes and correlations in the CSF have been reported, but due to difficulties in quantification and visualization, there have been few studies to determine the exact mechanisms related to pathophysiological changes in CSF of other neurological diseases [12,13,14]. Therefore, if the qualitative and quantitative mitochondrial changes in CSF after LSS are investigated, this will contribute to the fundamental understanding of changes in the microenvironment and development of a treatment method addressing the root cause. Therefore, we hypothesized that extracellular mitochondria within the CSF may be a potential prognostic biomarker for early detection of LSS and this study further opens up the possibility to develop a new type of extracellular-mitochondria-targeted therapy in CSF for LSS.

## 2. Materials and Methods

### 2.1. Rat LSS Model

Male Sprague–Dawley rats (7 weeks old, 230–250 g) were obtained from Daehan Bio Link (Chungju, Korea). All procedures were approved by the Jaseng Animal Care and Use Committee (approval no. JSR-2021-02-001-A). Rats were housed in standard cages under constant temperature (23–25 °C) and humidity (45–50%) with a 12 h light/dark cycle. The rats had free access to food and water. Animals were anesthetized with 2–3% isoflurane gas (Forane; BK Pham, Goyang, Korea), and a dorsal laminectomy was performed at L5 using fine rongeurs. Next, a silicone block (80 kPa, 4 × 1 × 1 mm^3^) was implanted at the L4 level using no. 5 fine forceps. Sham-operated rats underwent laminectomy at the L5 level, without implants. The spinal cord was then covered with Surgicel^®^ absorbable hemostat (Johnson and Johnson, Arlington, TX, USA). To prevent infection, all rats were injected intramuscularly with 40 mg/kg cefazolin sodium (Cefazolin^®^, Chong-Kun-Dang Pharm., Seoul, Korea) after suturing. All rats were also administered an oral dose of 10 mg/kg Children’s Tylenol^®^ (Janssen Korea Inc., Seoul, Korea) after anesthesia was resolved for pain management. Rats (*n* = 10 per group for each analysis) were sacrificed 1 week after silicone implantation.

### 2.2. Locomotor Function Assays

We used two tests to assess the quality and reliability of our animal models: the Basso, Beattie, and Bresnahan (BBB) scale, and the horizontal ladder test as described previously [15]. The BBB scale is expressed as a score from 0 to 21 points (no hindlimb movement was scored 0 and normal hindlimb movement was scored 21). Two independent observers analyzed hindlimb motion in an open field (cylindrical acrylic box; 90 cm diameter, 15 cm height) for 4 min. Average values were used. The ladder walking test was used to test the ability of the rats to maintain balance. All rats walked on a metal runway (2.5 cm between grids) from left to right three times, and their movements were captured with a digital camcorder. The score was calculated as follows: ladder score (%) = erroneous steps of hind limb/total steps of hind limb × 100. Locomotor functions were examined 7 days after the induction of LSS. All locomotor tests were recorded using a digital camera and were performed by two observers who were blinded to the treatments.

### 2.3. CSF Collection

**Figure 1:**
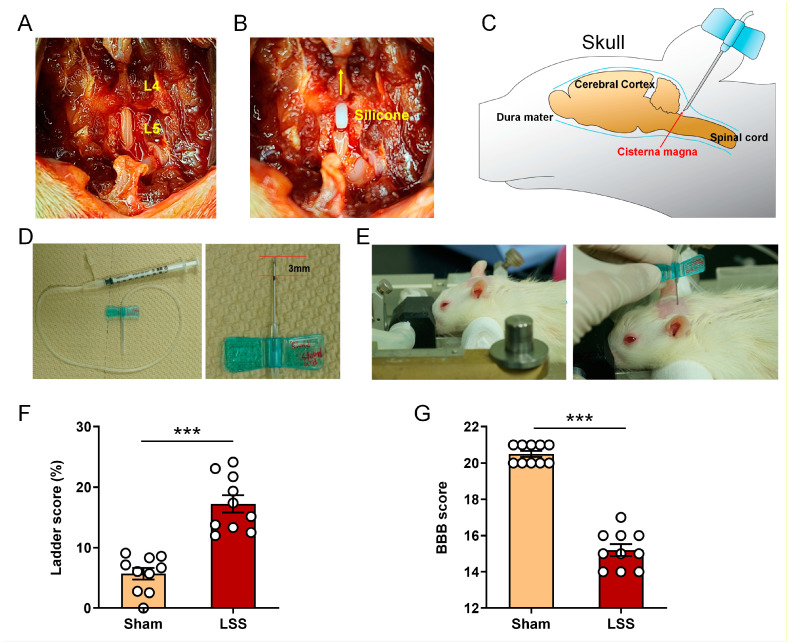
LSS surgical procedure and technical collection of CSF. (**A**) Surgical images after L5 laminectomy for making the LSS model. (**B**) Appearance of silicone implantation into L4 level through exposed L5 level using fine forcep. (**C**) Rat brain atlas to show the cisterna magna for CSF collection. (**D**) Construction of draw syringe and depth marking with three millimeter in the needle. (**E**) The position of rat’s head at approximately 45° and appearance of collecting the CSF from cisterna magna in rat. (**F**,**G**) Ladder (**F**) and BBB (**G**) assessment for confirm the standardized LSS model. Data are expressed as the means ± SEM. Significant differences indicated as *** *p* < 0.001 vs. the sham group were analyzed via unpaired *t* test with Welch’s correction.

The CSF was prepared in accordance with previously published protocols [16] and collected at 1 week after LSS. A 23-G scalp vein (Doowon MediTec, Co., Gimje, Korea) was connected to clear PE-50 tubing, and the other end of the tube was attached to a 1 mL syringe, as shown in Figure 1. Prior to CSF collection, rats were anesthetized with 2–3% isoflurane gas, and the neck region of each rat was shaved using an electric clipper. Then, the orientation of the animal’s head was maintained at approximately 45°. A depressible surface with the appearance of a rhomb between the occipital protuberance and the spine of the atlas became visible. A 23-G scalp vein connected to a draw syringe was horizontally and centrally inserted to collect CSF from the cisterna magna without making any incision in this region. The colorless CSF sample was slowly drawn into a syringe. When CSF no longer came out, altered resistance was easily felt in the process of pulling out slowly with the 1 mL syringe. Upon the appearance of blood contamination, the PE tubing was pinched off above the contamination site and the tubing was cut at this point. The non-contaminated sample was collected into 2 mL Corning Cryogenic Vials (Sigma-Aldrich, St. Louis, MO, USA) and stored in liquid nitrogen until analysis.

### 2.4. Flow Cytometry Analysis

A flow cytometric assay was used to investigate altered reactive oxygen species levels and endogenous extracellular mitochondrial changes within the CSF after LSS. The collected CSF should be analyzed immediately for flow cytometric analysis and kept in 37 °C on heating block (FINEPCR, Gunpo-si, Korea) during the time between sampling and analysis. Briefly, the collected 200 µL of CSF was centrifuged at 2000 rpm for 3 min. The supernatant was carefully removed using a pipette and collected in another tube. The cell pellet was resuspended in different staining solutions. The following four staining solutions were prepared as follows:

(A) Cellular ROS level detection solution: cellular ROS levels were measured in CSF using 2′,7′-dichlorofluorescin diacetate (DCFDA; Sigma-Aldrich, St. Louis, MO, USA). We prepared a 5 mM stock solution by dissolving 9.3 mg DCFDA powder in high-quality 3.8 mL anhydrous dimethylsulfoxide (DMSO) solution and prepared 1 mL of 10 µM DCFDA solution.

(B) MitoSOX solution: we prepared a 5 mM MitoSOX (Thermo Fisher Scientific, Waltham, MA, USA) stock solution by dissolving 50 µg of MitoSOX in 13 µL DMSO solution and prepared 5 µM MitoSOX working solution with 1 mL of a BSA stain buffer (BD Biosciences, Torreyana Rd., San Diego, CA, USA) for staining.

(C) Mitotracker solution: we prepared a 1 mM Mitotracker (Thermo Fisher Scientific, Waltham, MA, USA) stock solution in DMSO solution, and prepared 200 nM Mitotracker working solution with 1 mL of a BSA stain buffer. 

(D) TMRM solution: we prepared a 10 mM Tetramethylrhodamine, methyl ester (TMRM, Thermo Fisher Scientific, Waltham, MA, USA) solution by dissolving 25 mg of TMRM in 5 mL DMSO solution and prepared 10 nM TMRM working solution with 1 mL of a BSA stain buffer.

The staining solution (0.1 mL) was directly added to the cell pellet and immediately analyzed using flow cytometry (Accuri C6 Plus Flow Cytometer, BD Bioscience, Franklin Lakes, NJ, USA). The mean positive cell values, as determined via flow cytometry, were expressed as a percentage relative to the sham group.

### 2.5. DNA Extraction

Total DNA was extracted from 200 µL of CSF according to phenol-chloroform-thiocyanate guanidine (Sigma-Aldrich, St. Louis, MO, USA), as described in detail previously [17]. A 200 µL CSF sample from each group was mixed with 500 µL of phenol-chloroform-thiocyanate guanidine. Then, 300 µL of ice-cold chloroform (Honeywell, Burdick, and Jackson, Muskegon, MI, USA) was added to the mixture and centrifuged at 12,000 rpm for 15 min at 8 °C for homogenization. The supernatant was carefully removed and transferred to an appropriate tube containing 500 µL of cold absolute ethanol (Merck, Darmstadt, Germany), followed immediately by vortexing for 5 s. The mixture was centrifuged at 12,000 rpm for 15 min at 8 °C, and the supernatant was carefully discarded. The pellet was washed with 500 µL of ice-cold absolute ethanol. After centrifugation at 12,000 rpm for 15 min at 8 °C, the supernatant was discarded. The pellet was then dissolved in 30 µL of Tris-EDTA (TE) buffer (Bioneer Co., Daejon, Korea) at 65 °C for 30 min. Samples were stored at −20 °C until further analysis.

### 2.6. PCR-Based Analysis of mtDNA/nDNA Ratio

The mitochondrial DNA (mtDNA) concentration was analyzed quantitatively between a target mitochondrial gene and a reference nuclear gene (mtDNA/nDNA) using quantitative real-time PCR (qPCR), as described previously. Briefly, mtDNA/nDNA ratios were measured by quantifying the number of mtDNA molecules per nDNA molecule calculated by the classical ΔΔCt method used for qPCR analysis using the following primers: mitochondrial NADH-ubiquinone oxidoreductase chain 1 (mt-ND1) forward: 5′-CCGTCCTCCTAATAAGCGGC-3′ and mt-ND1 reverse: 5′-TATGGCTATTGGTCAGGCGG-3′; GADPH forward: 5′-CCCCCAATGTATCCGTTGTG-3′ and GAPDH reverse: 5′-TAGCCCAGGATGCCCTTTAGT-3′.

### 2.7. Statistical Analysis

All numerical data are expressed as mean ± standard deviation. Prism 9.0 (GraphPad Software, San Diego, CA, USA) was used for all analyses. Data are expressed as the mean ± SEM. Significant differences are indicated as * *p* < 0.05 or ** *p* < 0.01 vs. the sham group and were analyzed via unpaired t-test with Welch’s correction.

## 3. Results

### 3.1. LSS Surgical Procedure and Technical Collection of CSF

The inset of the surgical images illustrates the procedures performed. The first image from the left shows that the laminectomy was performed in the L5 vertebral lesion without nerve compression to push the silicone block into the L4 level (Figure 1A). A silicone block with 80 kPa stiffness was inserted underneath the L4 level (Figure 1B). The schematic illustrates the needle insertion site on the cisterna magna, as indicated in the images (Figure 1C). Subsequently, we performed sequential procedures for CSF collection (Supplementary Materials, Video S1). A 1 mL syringe with a 23 G scalp vein was prepared and marked manually at a 3 mm depth on the needle (Figure 1D). The rat’s head was fixed on a frame. A needle connected to a draw syringe was inserted horizontally and centrally in the cisterna magna between the occipital protuberance and the spine of the altlas (Figure 1E). All CSF samples were analyzed 1 week after LSS. In addition, the locomotor function was assessed using two methods (BBB and ladder tests) to determine whether the LSS model was made accurately and uniformly. The LSS group showed a significantly higher foot-slip frequency over the total number of steps by hindlimbs, whereas the sham group had a foot-slip rate of approximately 5 % in 1 week (Figure 1F). The BBB score of the LSS group decreased significantly after 1 week compared with that of sham group (Figure 1G). The sham group had an average score of 21 points and displayed consistent plantar stepping with consistent coordination. The LSS group had an average BBB score of 15 points in the first week, which is approximately six points lower than the average score of the sham group.

### 3.2. Flow Cytometric Cell Size and Number Analysis in CSF after LSS

**Figure 2:**
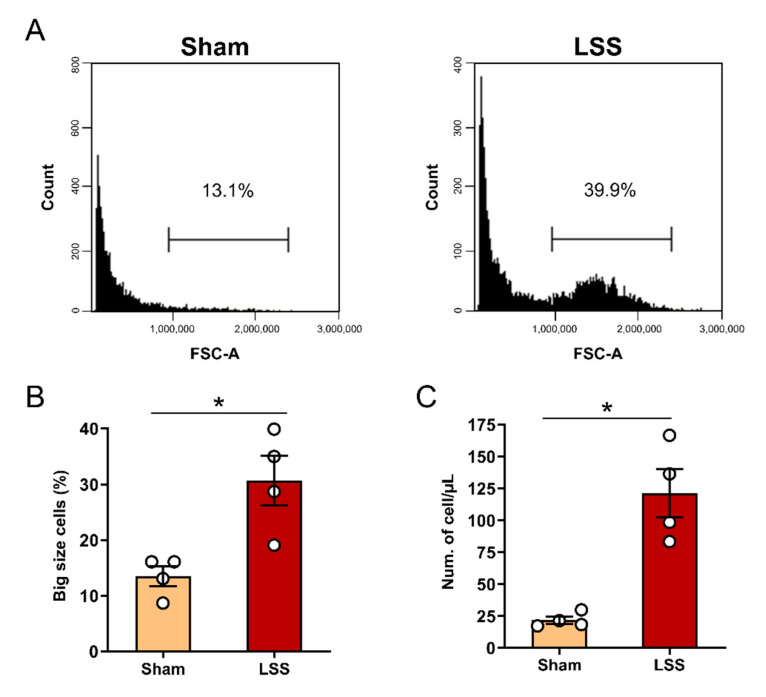
Flow cytometric cell number and size analysis in CSF after LSS. (**A**) Representative flow cytometry dot blot of the cell number and size in sham and LSS groups using the forward scatter (FSC) parameters. (**B**) Flow cytometric percentages of large cells and (**C**) number of cells per 1 μL within the CSF in sham and LSS groups. Data are expressed as the means ± SEM. Significant differences indicated as * *p* < 0.05 vs. the sham group were analyzed via unpaired *t* test with Welch’s correction.

To assess the changes in cell size and number within the CSF after LSS, we analyzed CSF collected from the sham and LSS models at 1 week by flow cytometry (Figure 2A). The quantification results showed that the cell size was significantly increased at 1 week after LSS (Figure 2B). We also quantified the total cell number within 200 µL of CSF. The total cell number showed a tendency to increase after LSS, and there was a significant difference between the two groups at 1 week (Figure 2C). Our findings revealed that an increase in the size and cell number was induced within the CSF by the given LSS.

### 3.3. Analysis of Extracellular Mitochondrial Mass within CSF after Given LSS

**Figure 3:**
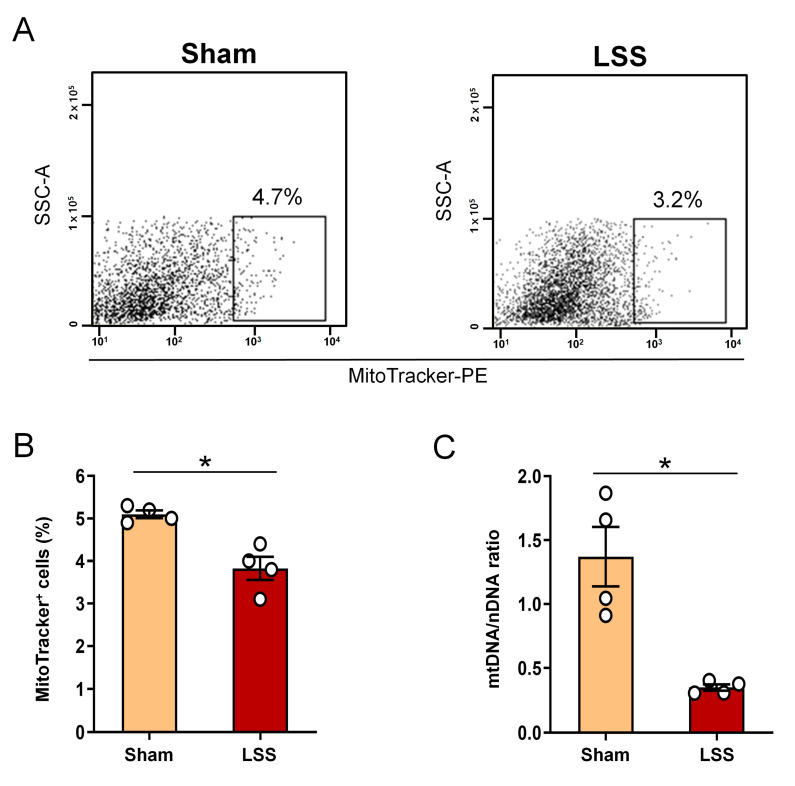
Analysis of extracellular mitochondrial mass within CSF after given LSS. (**A**) Representative flow cytometry dot blot of the MitoTracker Deep Red FM in sham and LSS groups. (**B**) Flow cytometric quantification of MitoTracker within the CSF in sham and LSS groups. (**C**) Real-time PCR based of mtDNA/nDNA ratio in sham and LSS groups. Data are expressed as the means ± SEM. Significant differences indicated as * *p* < 0.05 vs. the sham group were analyzed via unpaired *t* test with Welch’s correction.

We also examined changes in endogeneous extracellular mitochondrial mass within the CSF after LSS. To verify the mitochondrial mass within the CSF, MitoTracker red fluorescent dye (MTR) has been commonly used as a powerful tool to evaluate the mitochondrial mass within CSF using flow cytometry (Figure 3A). MTR positivity tended to decrease in comparison with the sham group and was significantly different between the groups (Figure 3B). We further quantified the mtDNA/nDNA ratio using qPCR (Figure 3C). The mtDNA/nDNA ratio was significantly lower in the LSS group than in the sham group and may reflect either a decrease in mitochondrial number/content or a depletion of mtDNA after LSS. Therefore, it is possible that the reduction in mitochondrial mass in the CSF is a marker of LSS.

### 3.4. Analysis of Cellular ROS and Mitochondrial ROS Production within CSF after LSS

**Figure 4:**
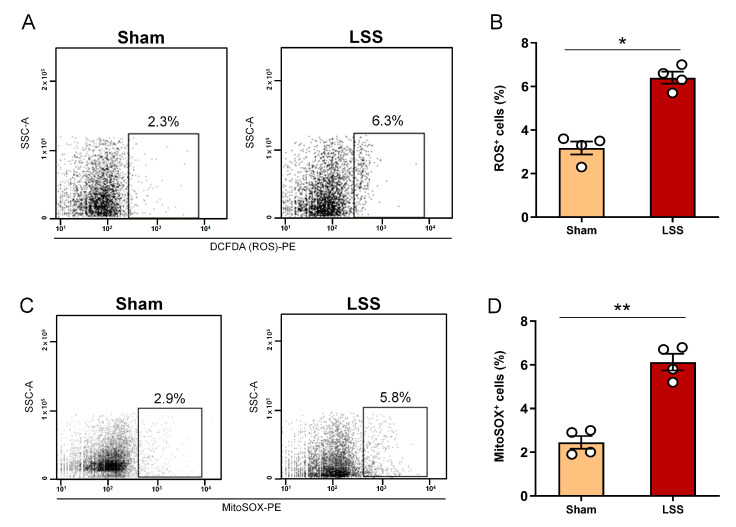
Analysis of cellular ROS and mitochondrial ROS production within CSF after LSS. (**A**) Representative flow cytometry dot blot of the ROS production in sham and LSS models using the FSC parameters. (**B**) Flow cytometric quantification of cellular ROS level within the CSF in sham and LSS groups. (**C**) Representative flow cytometry dot blot of the mitochondrial ROS (MitoSOX) in sham and LSS groups using the FSC parameters. (**D**) Flow cytometric quantification of MitoSOX within the CSF in sham and LSS groups. Data are expressed as the means ± SEM. Significant differences indicated as * *p* < 0.05 and ** *p* < 0.01 vs. the sham group were analyzed via unpaired *t* test with Welch’s correction.

We investigated ROS production and mitochondrial ROS (mtROS) changes within the CSF 1 week after LSS. ROS have long been known to cause oxidative modifications to changes in some cellular components. Mitochondria are the main sources of ROS within mammalion cells. Overproduction of ROS causes oxidative damage to lipids, proteins, and DNA, which has been implicated in various diseases in response to oxidative stress, including cancer and respiratory, cardiovascular, neurodegenerative, and digestive diseases. Thus, ROS quantification in the CSF has long been considered suitable for the diagnosis of various diseases. We detected oxidized CM-H_2_DCFDA (DCFDA) to confirm cellular ROS levels within the CSF using flow cytometry (Figure 4A). DCFDA is a cell-permeable fluorogenic probe that is used as an indicator of cellular ROS in cells. DCFDA positivity revealed a significant increase in ROS production in the LSS group compared with that in the sham group (Figure 4B). In addition, we analyzed whether mitochondrial oxidation in CSF was elevated after LSS by flow cytometry (Figure 4C). MitoSOX permeates live cells where it selectively detects superoxide (O_2_^−^) in the mitochondria. Previous studies have demonstrated that mitochondrial changes are closely associated with neurological diseases and were recently discovered within the CSF. We analyzed whether mitochondrial oxidation in CSF was elevated after LSS by flow cytometry, as shown in Figure 4D. MitoSOX fluorescence was significantly increased in the CSF after LSS compared to the sham group. We showed, for the first time, an association between LSS and both elevated cellular ROS and mitochondrial ROS levels in CSF.

### 3.5. Analysis of MMP within CSF after Given LSS

**Figure 5:**
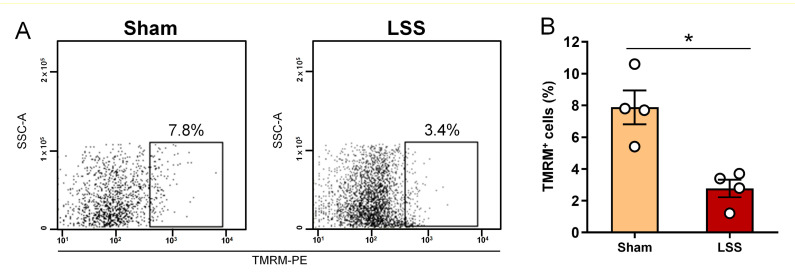
Assessment of MMP within CSF after given LSS. (**A**) Representative flow cytometry dot blot of the TMRM in sham and LSS models using the FSC parameters. (**B**) Flow cytometric quantification of TMRM within the CSF in sham and LSS models. Data are expressed as the means ± SEM. Significant differences indicated as * *p* < 0.05 vs. the sham group were analyzed via unpaired *t* test with Welch’s correction.

Next, we also confirmed whether the change in MMP in mitochondria in the CSF was causally related to LSS. TMRM is a cell-permeant dye that accumulates in active mitochondria with intact membrane potentials. Flow cytometric analysis of the CSF was performed to detect changes in TMRM 1 week after LSS (Figure 5A). TMRM positivity was significantly decreased at 1 week in the LSS group compared to that in the sham group (Figure 5B). Therefore, a lower MMP in mitochondria in the CSF was correlated with the onset of LSS and considered a potential biomarker for diagnosis.

## 4. Discussion

Oxidative stress has been proposed as an important pathophysiological mechanism in cardiovascular and degenerative diseases, as well as in neurological diseases, and excessive ROS production has emerged as the main cause [18]. Mitochondria are the key organelles producing ROS, and when mitochondria are damaged, resulting in morphological and functional changes, most of the oxygen is used to produce ROS [19]. Excessive ROS production causes DNA damage in cells and creates an oxidative stress environment that induces cell death and tissue damage. LSS is characterized by a narrowing of the spinal canal and nerve compression in the spine. A recent study with LSS reported that the deformity of the spine was due to oxidative stress caused by aging [20,21]. 

Aging is the result of an accumulation of oxidative aged population when cell homeostasis is not maintained. The accumulation of this oxidative damage to intracellular proteins and organelles is normally repaired by the homeostatic system, but when the function of this system is reduced with age, ROS production in aged mitochondria causes oxidative damage to organelles and macromolecules as by-products of numerous enzymatic reactions and consequently creates an oxidative stress environment that triggers cell death and tissue damage [22,23]. Therefore, the accumulation of oxidative proteins and free radicals due to failure to undergo proper proteolysis was suggested as the cause of many degenerative diseases related to aging. However, there is a lack of understanding of the mechanism for maintaining intracellular homeostasis from oxidative stress in spinal canal stenosis disease and research on therapeutic targets that fundamentally inhibit oxidative stress. There has been no research yet that directly analyzes the relationship between LSS, mitochondrial dysfunction, and oxidative stress, but new therapeutic strategies by targeting mitochondria in terms of the functional changes and correlation were presented in several neurological diseases such as spinal cord injury, multiple sclerosis, and parkinson’s disease [24,25,26]. In addition, mitochondria are the only animal organelles that have their own DNA, except for the nucleus. Recent studies have reported that CSF contains cell-free mtDNA and that changes in mtDNA concentration are associated with neurodegeneration [27,28]. Therefore, it has been reported that concentration changing of CSF mtDNA affects the process and progression of a disease and is related to molecular factors related to damage causing an inflammatory response. In addition, by analyzing the difference in MMP in mitochondria in the CSF, it was confirmed that the MMP showed a significant decrease compared to the normal level with disease progression. Therefore, endogenous extracellular mitochondria in CSF are expected to serve as biomarkers that can indirectly reflect changes in the central nervous system and provide the basis for a new treatment strategy. In this study, we analyzed ROS, mtROS, and MMP in the CSF of LSS rats. The major finding of this study is that concentrations of free circulating mtDNA are decreased in CSF of LSS group compared with sham group. Additionally, a significant increase of oxidation was observed in CSF with LSS. Particularly, the extracellular mitochondria of CSF represents an increase of mitochondrial superoxide, and the membrane potential, an index related to the function, was also significantly decreased.

These preliminary findings suggest that altered levels of ROS, mtROS, and MMP in CSF are pathophysiologic phenomena for understanding and treating LSS and are potential biomarkers for the diagnosis of LSS. However, we do not know CSF pathophysiological changes in the chronic stages of LSS. Additionally, there have been few studies on the relationship between the increase in mitochondrial oxidative stress in the CSF and neuronal injury, pain, and behavioral changes with LSS. 

Based on this study, with further research on the relationship between the quantitative and qualitative changes of mitochondria in CSF, pathophysiological mechanisms, and functional changes occurring after a given LSS, the mitochondria in CSF are expected to be useful indicators for the early diagnosis and development of treatment methods for LSS.

## 5. Conclusions

In this study, we found that ROS and mtROS production was increased in the CSF of LSS rats, and MMP was also decreased in CSF after LSS. We concluded that endogenous extracellular mitochondrial changes in the CSF might contribute to LSS-induced pathophysiological processes. Therefore, assessment of mitochondrial changes in the CSF may indirectly provide a way to diagnose LSS, adding a promising and complementary strategy to the treatments of LSS.

## Data Availability

The data presented in this study are available on request from the corresponding author.

## Supplementary Materials

The following are available online at https://www.mdpi.com/article/10.3390/diagnostics11050748/s1. Video S1: Technical procedure for CSF collection from the cisterna magna.
